# Disaster risk reduction in Iraq

**DOI:** 10.4102/jamba.v11i1.656

**Published:** 2019-02-25

**Authors:** Mustafa T.M. Al-Shamsi

**Affiliations:** 1 Unit of Investigation in Emergency and Disaster, University of Oviedo, Oviedo, Spain; 2 Center for Research on the Epidemiology of Disasters, The Catholic University of Louvain, Belgium

## Abstract

Iraq is at risk of multiple hazards including both natural and man-made calamities. Little effort had been made before 2003 to address the disaster risk; even though many legislations enacted to provide a relief in the event of the acute crisis, they were mainly focused on the reactive response to the calamities without taking into consideration the prevention, preparedness and mitigation approach. The recent years have witnessed some positive attitude from the government and international society to develop strategies for disaster risk reduction in Iraq. Iraq for the first time has drafted a law that is distinctive for the disasters. The purpose of this article is to review the possibilities and challenges of disaster risk reduction in Iraq.

## Introduction

Iraq is at risk of multiple disasters ranging from the natural disasters such as drought, sandstorm, heatwaves, floods, desertification and epidemics to man-made ones. This susceptibility accompanied by the effect of wars and conflicts in the last four decades results in multiple hazardous effects such as contamination of the land by biological materials, unexploded ordnance and cluster munitions. Wars and conflicts affected the functionality of the system to respond to disasters. This, combined with the effect of poverty and displacement, results in increase in the vulnerability of the people and further compromises the system leading to many dysfunctional services. Based on our specialisation in disaster, we have included a review of the current situation of disaster risk reduction (DRR) in Iraq. The objective of this article is to give an account on the current framework for DRR strategies in Iraq. Because of scarce resources on this subject, we have tried to review the national laws and newspapers as well as the United Nation Development Programme (UNDP) agenda, which is the main supporter of the implementation of DRR in Iraq.

## The strategies and the legislative context of the disaster risk reduction in Iraq

On the first session of the Global Platform for Disaster Risk Reduction conference held at Geneva in 2007, the director of the Iraqi Ministry of Environment stated that the Iraqi government is making every effort to decrease the natural and man-made disasters in the country (Iraq statement 2007). After 2003, Iraq became more actively involved in the strategies of DRR. In 2007, Iraq established the Natural Disaster Risk Committee. This committee coordinates the response to natural and man-made disasters through preparedness, prevention and mitigation approach according to the *Hyogo* Framework for Action. Moreover, Iraq, for the first time, becomes part of the regional DRR programme. In 2008, Iraq indorsed the Arabian Center for Earthquake Hazard and Natural Disaster Prevention programme. The aim of this programme is to form a system for the countries that share common borders to enable them to respond better to the natural calamities. According to this project, Iraq is committed to show scientific and technical cooperation with the other Arab countries in order to prevent, prepare, mitigate and respond to the effect of natural disasters. Furthermore, the mission of Iraq includes providing logistical and financial support, executing studies to evaluate the natural hazards, sharing the information with other centres in the region and mapping earthquakes and natural hazards.

In terms of disaster response, the Iraqi government has always followed the reactive pattern. In other words, it follows the response and relief approach. There are several legislations issued to mitigate the suffering of people in case of disasters and sudden humanitarian emergencies such as the Emergency Use Law 1961, Civil Defence Law 1978, Public Health Law 1981 and Social Care Law 1980. Additional laws that have been enacted after 2003 include the Immigration Law, which deals with the issues of internally displaced people; the Governorate Law, which regulates the response between the governorate and central authority in case of disaster; and the Law of Budget Management, which coordinates the allocation of financial resources to disaster-affected area.

On an institutional level, the Iraqi government has established several institutions to respond to the crisis after 2003. This includes Governorate of Emergency Cell (GEC) which is composed of the representatives of the Directorate of Public Health Division, Ministry of Migration and Immigrant, the local Non-Governmental Organizations (NGOs) and the United Nations Office at the governorate. In response to any disastrous situation, the GEC of the affected governorate may respond in a decentralised pattern. Should a disaster occur beyond the capacity of the GEC of the affected governorate, the governor then has the right to call for the central government authority. Thereafter, a higher coordination committee may be formed under the patronage of the prime minister to manage the province that faces an emergency situation.

The most critical centre is the National Operation Center (NOC), which is operated directly by the prime minister’s office; it is usually specialised in terror attacks. Another centre that is established with the escalation of 2006 violence is the National Crisis Action Cell (CAC). The main mission of CAC is providing a national level crisis management, and CAC has the authority to coordinate between the ministries. Furthermore, the Inter-Ministerial Committee on Disaster Management (IMCDM) is another body created in 2007. It is composed of ten ministries including the Interior, Defence, Planning and Development, Health, Communication, Environment, Water Resources, Foreign Affair, Science and Technology as well as the State Ministry of National Security and the Secretariat General of Council Ministers. The IMCDM had prepared notes to establish the National Center for Disaster Management. The centre’s mission is to act as a secretariat for DRR actions including research and studies on emergency, planning and coordination of intervention in case of acute crisis. Additionally, it has the role of information exchange and providing a database for recording the information on hazards, risk and vulnerability of disasters and acute crisis situations.

## Disaster risk reduction so far

The Ministry of Science and Technology (MOST) is the focal point for the coordination between the ministries regarding the response of disasters in Iraq. Under the patronage of MOST, the first Disaster Risk Reduction and Mitigation Law has been drafted and submitted for approval by the parliament in 2012 with the help of UNDP (UNDP 2013, 2014). The law complies with the Arab Strategy of Disaster Risk Reduction and is approved by the United Nation Disaster Risk Reduction framework. Under this law, a number of institutions were proposed to be created such as Disaster Reduction Councils and National Disaster Risk Reduction and Management Center. The law provides a comprehensive approach to address the risk of natural and man-made disasters in the country through early warning, preparation and response system. The institutions that support the implementation of this law are the Multi-Sectoral National Disaster Committee which is led by the Ministry of Environment and the National Center for Disaster Risk Reduction. The disaster risk plan is composed in accordance with the *Hyogo* Framework for Action 2005–2015.

However, on the final report of the Ministry of Environment, the Iraqi government has failed to implement the strategic steps of the *Hyogo* Framework (Hyogo 2015). This is because of poor governance and negligence from both national and local authorities. Moreover, no specific budget has been allocated to support resilience programmes and build infrastructures which improves the response to disasters. The main challenges are the poor coordination between the government and the agencies that are responsible for addressing the risk of disasters. Another challenge is the absence of national criteria to define the risk of the disaster on the country level. Although there was a good warning system according to the report, further bolstering of the alert system was recommended. In the final national progress report, the Ministry of Environment has placed a future plan for the establishment of National Disaster Management Center as well as working on secure funding for the activities that related to disaster management (Hyogo 2014).

The last report on the implementation of the *Sendai* Framework was in 2017. Unfortunately, Iraq still lack the national database programme to conduct disaster losses. The precise statistical measures about the possible areas and vulnerable people to disaster are still underdeveloped. Nevertheless, the Iraqi officials claim that a national programme for DRR is still in the preparation phase (Sendai 2017).

## Conclusion

Iraq still follows the traditional form of response to emergency with no prior preparedness to such events. This is partly because of poor governance and lack of awareness to the importance of DRR strategies and partly because of the fact that natural disasters were not frequent in recent years. Thus, despite the legislation of multiple laws, it remains inapplicable in the reality ground. The general institutional context of the disasters still lacks essential communication and early warning systems. Unfortunately, the draft of the first Disaster Risk Reduction Law has many flaws such as failure to define the type of disasters and disaster contingency planning that includes disaster mapping and vulnerability assessment. Additionally, the National Disaster Law only provides a framework for the national disaster plan; however, it needs to be complemented with by including disaster management and emergency preparation plan. This approach is performed by preparing either a separate regional and national plan as in Sri Lanka (Sri Lanka 2005) or a single plan that covers pre- and post-disaster phases such as the New Zealand National Civil Defence Emergency Management Plan (New Zealand 2002). Furthermore, the law does not precisely define the role of the local authorities in the disaster cycle. This is in contrast to the *South African Disaster Management Act* that provides clear classification and declaration of disaster at both local and national levels (South Africa 2002).

The main issue is that the existing institutional arrangement at national, regional and local levels is focusing on the post-disaster action without taking any consideration for preparing for a pre-disaster phase such as prevention and mitigation planning. Therefore, Iraq is still lacking comprehensive disaster management systems based on analysis of risk, hazards, capacities and vulnerabilities of the affected population. Seemingly, Iraq needs strong infrastructural and technical capacities within the government and other stakeholder bodies to be able to respond to the future crisis. War and post-war conflict destroy infrastructures and leave the majority of institutions dysfunctional, thereby increasing the vulnerability of the population. Moreover, the poverty and displacement further make the country unable to respond to disasters. Although the international NGOs have a vital role in this response, they have no capacity to support complete disaster reduction and mitigation planning in Iraq without the effort of the local government. Nevertheless, overall, Iraq is making some steps in the right direction towards DRR and response, and is slowly progressing.
